# Architecture and enhanced-algorithms to manage servers-processes into network: a management system

**DOI:** 10.7717/peerj-cs.1408

**Published:** 2023-07-18

**Authors:** Fayez AlFayez

**Affiliations:** Computer Science and Information, College of Science in Zulfi, Majmaah University, Al-Majmaah, Saudi Arabia

**Keywords:** Scheduling, Network, Heuristic, Decision-tree, Server-execution

## Abstract

This work investigates minimizing the makespan of multiple servers in the case of identical parallel processors. In the case of executing multiple tasks through several servers and each server has a fixed number of processors. The processors are generally composed of two processors (core duo) or four processors (quad). The meaningful format of the number of processors is 2^*k*^, and *k* ≥ 0. The problem is to find a schedule that minimizes the makespan on 2^*k*^ processors. This problem is identified as NP-hard one. A new network architecture is proposed based on the addition of server management. In addition, two novel algorithms are proposed to solve the addressed scheduling problems. The proposed algorithms are based on the decomposition of the main problem in several sub-problems that are applied to develop new heuristics. In each level of the generated tree, some results are saved and used to decompose the set of processes into subsets for the next level. The proposed methods are experimentally examined showing that the running time of the proposed heuristics is remarkably better than its best rival from the literature. The application of this method is devoted to the network case when there are several servers to be exploited. The experimental results show that in 87.9% of total instances, the most loaded and least loaded subset-sum heuristic (*MLS*) reaches the best solution. The best-proposed heuristic reaches in 87.4% of cases the optimal solution in an average time of 0.002 s compared with the best of the literature which reaches a solution in an average time of 1.307 s.

## Introduction

The application of network scheduling is exploited in several research works (Jemmali, Melhim & Al Fayez, 2022; Sarhan, Jemmali & Ben Hmida, 2021; Alquhayz & Jemmali, 2021; Jemmali & Alquhayz, 2020). Especially, the problem of treating the scheduling of processes on identical parallel processors is widely investigated in computer science. It can be defined by giving *n* processes (jobs) *J* = {*J*_1_, *J*_2_, …, *J*_*n*_} and *n*_*P*_ identical parallel processors *Pr* = {*Pr*_1_, *Pr*_2_, …, *Pr*_*n*_*P*__}. Each process *J*_*j*_ has an associated time *p*_*j*_ with *j* = {1, 2, …, *n*}. We assume that each *p*_*j*_ is a positive integer and 1 < *n*_*P*_ < *n* to avoid trivialities. A processor can execute at most one task at a given time. In addition, the task can not be processed by more than one of the *n*_*P*_ processors. The preemption of processes is not allowed for this study. This work mainly focuses on minimizing the maximal completion time of processes (makespan) in literature this problem is denoted as *Pm*||*C*_*max*_ (Graham et al., 1979).

A solution for *Pm*||*C*_*max*_ is presented by a set *S* = {*S*_1_, *S*_2_, …, *S*_*n*_*P*__} of the set *J*, where each *S*_*i*_ is the subset of processes scheduled on the processor *Pr*_*i*_. The workload of *Pr*_*i*_ is denoted by *C*(*S*_*i*_) where *C*(*S*_*i*_) = ∑_*j*_*k*_∈*S*_*i*__*p*_*k*_. This means that *C*(*S*_*i*_) is the completion time of the latest process scheduled on the processor *Pr*_*i*_. For each assignment *S*, set *C*_*max*_(*S*) = max_*Pr*_*i*__{*C*(*S*_*i*_)} which represents the makespan related to the solution *S*. This problem is important in practice because the objective is to seek the balancing of the load over the various processors, which is corresponding to finishing all processes in a minimum time and having a good distribution on processors. The studied problem is an intensively studied one in scheduling that has remarkable practical interest and significant utilization in our life applications. *Pm*||*C*_*max*_ is *NP*-hard problem in the strong sense, see (Garey & Johnson, 1979). Several researchers applied the parallel machine problem to solve other related problems like learning effect constraint with minimization of the makespan (Jemmali & Hidri, 2021; Hidri & Jemmali, 2020), mold constraints (Hmida & Jemmali, 2022) or flow shop problem (Jemmali & Hidri, 2023; Amdouni et al., 2021; Agrebi et al., 2021; Jemmali et al., 2021). It is important to find an approximate solution for a problem that is classified as a hard one. The algorithms that solve the parallel machine problem can be applied to several industrial problems. The wide utilization of this well-known problem makes the study of this problem more imposing. In the literature, there are several works that solve the problem optimally but the time for some instances is time-consuming or in several cases does not reach the optimal solution. This article investigates the problem of scheduling algorithms to propose a new procedure to enhance the approximate solution performance.

The problem of *Pm*||*C*_*max*_ is widely investigated in the literature. One of research directions was conducted to develop exact solution methods and to show the lower bounds and heuristics. For instance, exact solutions have been studied and investigated in Mokotoff (2004), DellAmico et al. (2008) and Haouari & Jemmali (2008). Additionally, many algorithms were developed by using constructive schema, a worst-case performance ratio is given, for *Pm*||*C*_*max*_ in Hoogeveen, Lenstra & Van de Velde (1997), Mokotoff (2001) and Phillips et al. (1998). In DellAmico et al. (2008), the authors presented a meta-heuristic and an exact solution using the existing dataset proposed in the literature review.

For multi-fit and multi-subset solutions based on subset-sum and bin packing problems (DellAmico & Martello, 1995). Developed results were demonstrated following performance procedures and lifting heuristics in DellAmico et al. (2008), Haouari, Gharbi & Jemmali (2006b) and Haouari & Jemmali (2008). An effective simulated annealing algorithm was developed to generate the near-optimal solution (Lee, Wu & Chen, 2006). Another study of *Pm*||*C*_*max*_ problem was proposed using a hop-field type dynamical neural network to find a solution for this NP-hard problem even for the case of two machines (Akyol & Bayhan, 2006).

A dual feasible solution method to solve the problem of the parallel machine with minimization of the makespan is studied in Haouari, Hidri & Jemmali (2008). Other research work is also treated for the same problem when authors proposed several lower bounds for the studied problem (Haouari, Gharbi & Jemmali, 2006a).

An application of scheduling problems using parallel processors on network domains and its application when there are several servers to be exploited such in railway monitoring domain, new solutions are developed in AlFayez, Melhim & Jemmali (2019), AlFayez (2020) and Jemmali, Melhim & Al Fayez (2022). Budgets balancing strategies algorithms are proposed by applying parallel processors in Jemmali (2019b), Alharbi & Jemmali (2020) and Jemmali (2019a).

In Mokotoff (1999), the authors present an approximation algorithm based on linear programming formulations with binary variables for decision. Additionally, heuristic algorithms that iteratively utilize *MF* and *LPT* rules on several jobs and machine sets, obtained by using the existing solution, have been proposed in the literature. These algorithms give a solution for the well-known problem of multiprocessor scheduling (Kuruvilla & Paletta, 2015). An approximate solution based on balancing the hop-field was proposed in Habiba et al. (2018). Other works using local-search methods with the utilization of partial solutions and mixing were presented in Paletta & Vocaturo (2011).

Another domain of application of the parallel processors is used in the gas turbines problem in Jemmali et al. (2019). The dispatching rules variants of the fair distribution of the used space in the cloud are proposed in Alquhayz, Jemmali & Otoom (2020). The latter work is an application of the parallel processor’s problem. Recently, several new applications of the scheduling problem are studied in Jemmali et al. (2022), Jemmali, Melhim & Al Fayez (2022) and Jemmali (2022).

The proposed algorithms in El-Soud et al. (2021) can be applied to the studied problem with applying new constraints. In the same context the proposed algorithms can be used the problems given in Hidri & Jemmali (2020), AlFayez et al. (2019) and Melhim et al. (2020), Melhim, Jemmali & Alharbi (2019), AlFayez (2023) and Melhim, Jemmali & Alharbi (2018).

The next section reviews the best existing heuristics for the *Pm*||*C*_*max*_. Assuming that *p*_1_ ≤ *p*_2_ ≤ ⋯ ≤ *p*_*n*_, we present three heuristics from the literature review. The first is thee longest processing time (*LPT*), which is the oldest one. Two other best-performed heuristics the multi-start subset-sum-based improvement heuristic (*MSS*) and the multi-start knapsack-based improvement heuristic (*MSK*) are also reviewed. Later, we compare the performance of our new technique with these three heuristics.

The article is structured as follows. Section 2 is reserved for the presentation of the best heuristics from literature. Section 3 details the proposed network architecture. In Section 4, the proposed heuristics are detailed. The experimental results are discussed in Section 5. Finally, the work is concluded in Section 6.

## Best Heuristics from the Literature

In this section, we present the best heuristics from the literature. These heuristics will be compared to the proposed ones. The heuristics of the literature that used for comparison are studied in Haouari, Gharbi & Jemmali (2006b).

### Longest processing time heuristic (*LPT*)

The processes are arranged in the non-increase arrangement of their processing times and scheduled on the parallel processors according to this arrangement. The first available processor is chosen to assign the process.

### A multi-start subset-sum-based improvement heuristic (*MSS*)

As shown in Haouari, Gharbi & Jemmali (2006b) the *P*_2_||*C*_*max*_ could be reformulated as a subset-sum problem. Pisinger (2003) introduced a pseudo-polynomial to solve a subset-sum problem using a dynamic programming algorithm. Based on this idea a multi-start local search algorithm was implemented.

### A multi-start knapsack-based improvement heuristic (*MSK*)

The *MSK* heuristic has a similar idea as (*MSS*). However, the main difference is localized in the problem solved in each procedure. Indeed, for *MSK*, each iteration solves a knapsack problem (*KP*) instead of the subset problem (*SSP*). Pseudo-polynomial time can solve *KP* efficiently.

## Proposed Network Architecture

**Figure 1:**
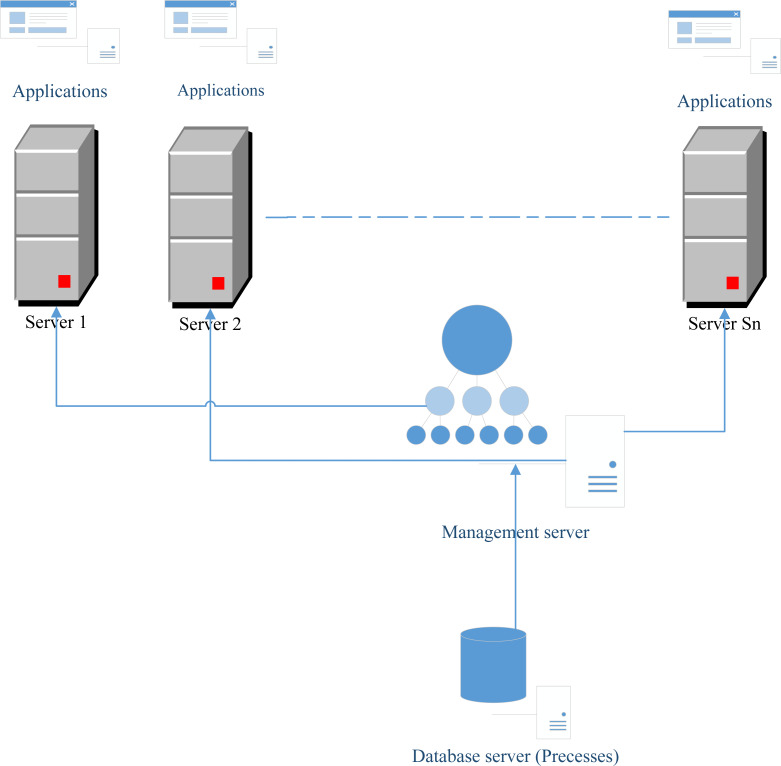
Proposed network architecture.

In this section, we propose a novel architecture that can ameliorate the execution of all processes using the scheduling problem. This architecture is based on the component that can call the best algorithm proposed in this article to solve a scheduling problem. This component is called the “Management server” (See Fig. 1). Firstly, the component “Database server” (as shown in Fig. 1) contains all tasks that must be executed. These tasks will be managed by the component “Database server” by applying a scheduling algorithm that solves the proposed problem to schedule these tasks to the different servers. It is worth noting that, each server contains a server number of processors and a different number of applications to run. Suppose that we have *Sn* servers. The problem is to find a method that we can schedule all tasks stored in the “Database server” component to the *Sn* servers. This problem is NP-hard and refereed to *Pm*||*C*_*max*_.

Figure 1 shows that in each server there are different applications to be launched. The number of servers constitutes a resource that is rare and the number will be limited because of the high cost of these resources. However, the number of processes is very big and must be executed in many cases in parallel. In this article, we add functionality to the “Management server” which is the calling of the best scheduling algorithm in order to address the problem of assignment. After calling of this algorithm, each process will know the server and the processor that will execute this process. In fact, each server has a fixed number of processors and each processor is identified by the variable *p*.

## Proposed Heuristics for 2^*k*^ Processors

In this section, we develop a new vision in order to select the number of processors based on the best utilized in the domain of computers. Indeed, we witness rapid development in computer hardware that utilizes more performance processors, for instance, core duo (two processors) and quad (four processors). Based on this idea, we refer to our approach to choosing a number of *n*_*P*_ = 2^*k*^ processors for the *P*||*C*_*max*_. Note here that in general 2^*k*^ ≤ *n*.

**Figure 2:**
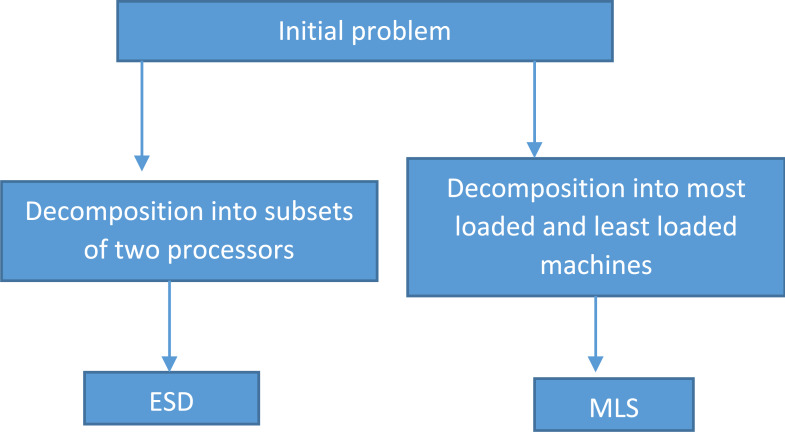
Flow chart of the proposed heuristics.

This article proposes two iterative heuristics. The first one is established on solving iteratively a number of *P*2||*C*_*max*_ problems using *SSP*. The second one is to reschedule the least and most charged processors. For the last step, a randomized heuristic will be applied. Figure 2 illustrates a flow chart for the proposed heuristics.

### An encapsulated subset-sum decomposition heuristic (*ESD*)

This heuristic generates an approximate solution using a binary tree method by solving a classification of two processor problems using subset-sum problems. The proposed heuristic *ESD* derived from the proposition given by Haouari, Gharbi & Jemmali (2006b) which shows that all problems of *P*||*C*_*max*_ where *m* = 2 written as *P*2||*C*_*max*_ will be written using the subset-sum problem. Indeed, the two processors are identical and parallel (*Pr*_1_ and *Pr*_2_). Suppose that the *Pr*_2_’s total workload does not exceed *Pr*_1_’s. Therefore, solving *P*2||*C*_*max*_ needs to minimize the workload of *Pr*_1_. Let *y*_*j*_ be a binary variable that takes the value 1 if the process *j* is scheduled to *Pr*_1_, and 0 otherwise. Thus, *P*2||*C*_*max*_ is formulated to solve:

(1) \begin{eqnarray*} \left\{ \begin{array}{@{}ll@{}} \displaystyle \min \nolimits {\sum \nolimits }_{{J}_{j}\in J}{p}_{j}{y}_{j},&\displaystyle \\ \displaystyle \text{s. t.}{\sum \nolimits }_{{J}_{j}\in J}{p}_{j}{y}_{j}\geq {\sum \nolimits }_{{J}_{j}\in J}{p}_{j}(1-{y}_{j}),\\ \displaystyle {y}_{j}\in \{ 0,1\} ,\forall {J}_{j}\in J. \end{array} \right. \end{eqnarray*}

In System 1, we replace ∑_*J*_*j*_∈*J*_*p*_*j*_*y*_*j*_ ≥ ∑_*J*_*j*_∈*J*_*p*_*j*_(1 − *y*_*j*_) by $ \left\lceil \frac{{\sum }_{{J}_{j}\in J}{p}_{j}}{2} \right\rceil $ and the obtained formulation is the subset-sum problem.

Finally, the system will be as follows:

(2) \begin{eqnarray*}SSP1: \left\{ \begin{array}{@{}ll@{}} \displaystyle \min \nolimits {\sum \nolimits }_{{J}_{j}\in J}{p}_{j}{y}_{j},&\displaystyle \\ \displaystyle \text{s. t.}{\sum \nolimits }_{{J}_{j}\in J}{p}_{j}{y}_{j}\geq \left\lceil \frac{{\sum \nolimits }_{{J}_{j}\in J}{p}_{j}}{2} \right\rceil ,\\ \displaystyle {y}_{j}\in \{ 0,1\} ,\forall {J}_{j}\in J. \end{array} \right. \end{eqnarray*}

where *y*_*j*_ takes 1 if process *J*_*j*_ is scheduled on the first processor, and 0 otherwise. Now, having the number *n*_*P*_ = 2^*k*^, we start by solving the problem of *n*_*P*_ processors and *n* processes applying the *SSP*1 for *P*2||*C*_*max*_. The solution decomposes *J* into two sets the first *J*_1_ and the second *J*_2_. Now, we treat *J*_1_ as a sub-problem with two processors solving by *SSP*1. Similarly, we solve the sub-set *J*_2_ by the same solving method. These solutions give new sub-set problems decomposed into processors and so on until arriving at the 2^*k*^. The Example 1 gives a more clear idea of the proposed heuristic.

**Table 1:** Instance with *n* = 10 and *n*_*P*_ = 4 applying *ESD*.

***j***	**1**	**2**	**3**	**4**	**5**	**6**	**7**	**8**	**9**	**10**
***p*_*j*_**	71	29	28	85	76	87	99	71	88	48

**Figure 3:**
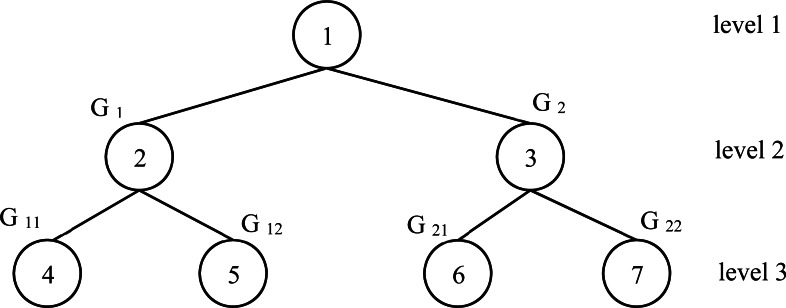
Fully complete tree for the example 1 instance.

Example 1 Let an instance with *n* = 10 and *n*_*P*_ = 4. The processing time for each process is given in Table 1. To apply the proposed heuristic *ESD*, the first step is the decomposition of all processes into 2 groups by applying the Subset-sum problem method. To do that, we calculate the summation of all processing time which is equal to 682. The two groups *G*_1_ and *G*_2_ is obtained by solving *P*2||*C*_*max*_ applying *SSP*1 with respect the capacity $C= \frac{682}{2} =341$. The corresponding solution is *G*_1_ = {71, 29, 28, 76, 88, 48} and *G*_2_ = {85, 87, 99, 71} Now, we repeat the same idea to *G*_1_ and *G*_2_. For *G*_1_, *C* = 170. Solving a *P*2||*C*_*max*_ for *G*_1_, we have two new groups denoted by *G*_11_ and *G*_12_. The corresponding solution is *G*_11_ = {29, 88, 48} and *G*_12_ = {71, 28, 76}. For *G*_2_, *C* = 171. Solving a *P*2||*C*_*max*_ for *G*_1_, we have two new groups denoted by *G*_21_ and *G*_22_ with *G*_21_ = {99, 71} and *G*_22_ = {85, 87}. Finally, it’s clear to see that we have a fully complete binary tree with three levels as shown in Fig. 3. As shown in Fig. 3 above, in the last level of the tree, we have the following groups *G*_11_, *G*_12_, *G*_21_ and *G*_22_ with the respective total completion times 165, 175, 170 and 172. Each group represents a processor for our studied problem. Applying this correspondence, we have:

• *G*_11_ is corresponding to *Pr*_1_
• *G*_12_ is corresponding to *Pr*_2_
• *G*_21_ is corresponding to *Pr*_3_
• *G*_22_ is corresponding to *Pr*_4_

Therefore, *C*_*max*_ = *max*{165, 175, 170, 172} = 175.

Theorem 1 A feasible solution of *P*||*C*_*max*_ problem with *n*_*P*_ = 2^*k*^, conduct the generation of a fully complete tree ( *FCT*) with the highest level equal to *k* + 1 and each node in the leaves in $ \left[ {2}^{l-1},{2}^{l}-1 \right] $ constitute one processor.

Proof The decomposition of the problem into a subset of 2 processor problems implies the division of the number of processes into two groups. Group for the first processor and another group for the second one. We continue the decomposition into two groups until reach level l. It is observable that the decomposition into two groups repetitively constructs a fully complete tree. The nodes in the leaf will be indexed in the interval $ \left[ {2}^{l-1},{2}^{l}-1 \right] $ which every node represents a processor. Now, drawing the (*FCT*) corresponding to the initial problems in order to solve by classing 2 processors in a sub-problem. This means that the feasible solution is encapsulated in (*FCT*).

**Figure 4:**
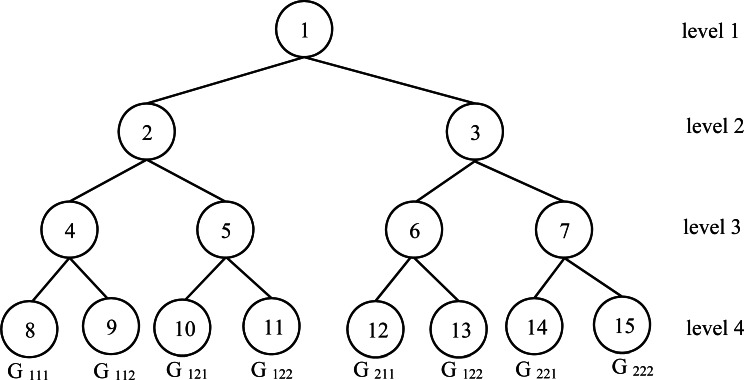
Encapsulated *FCT* for feasible solution search.

Example 2 Let the number of processors as *n*_*P*_ = 2^3^ = 8. In this case, the number of levels for the ( *FCT*) is 4. The last level contains 8 nodes. The index of these eight nodes is in $ \left[ {2}^{l-1},{2}^{l}-1 \right] =[8,15]$. These indexes will constitute the corresponding processors for the initial problem with *n*_*P*_ = 8. Figure 4 illustrates the encapsulated *FCT* for feasible solution search.

### Most loaded and least loaded subset-sum heuristic *MLS*

The idea of this heuristic can be explained in the following steps. In the first, we apply the *ESD* heuristic described above. From the schedule given by the *ESD* heuristic, we fix the highest load processor *Pr*_1_ and the lowest load processor *Pr*_2_. Applying the subset-sum problem with *P*2|*C*_*max*_ we obtain the new distribution of processes on *Pr*_1_ and *Pr*_2_. This distribution consists of the newly obtained schedule with the enhanced *C*_*max*_ which constitutes *MLS*.

## Experimental Study

In this section, we highlight and analyze the results of the execution of our implementation. In order to examine the performance of the new proposed heuristics, we coded all algorithms in Microsoft Visual C++ (Version 2013). All the programs were tested on an Intel core i7 CPU 1.8 GHz personal computer with 8GB RAM using Windows 7 operating system with 64 bits. The heuristics were tested on several instances in order to obtain a good analysis of the performance. We adopt the way of generation of the processing time that is described in DellAmico & Martello (1995). Two distribution was applied. The first one is the discrete uniform distribution denoted by *U*[.]. The second one is the normal distribution denoted by *N*(.). Five classes are generated to show the experimental results as follows.

• *Class*1: *U*[1 − 100];
• *Class*2: *U*[20 − 100];
• *Class*3: *U*[50 − 100];
• *Class*4: *N*(100, 50);
• *Class*5: *N*(100, 20).

For each *Class* and for each pair of *n*_*P*_ and *n*, we generate 10 instances, which result in 2,350 instances in total. The choice of the pair (*n*, *n*_*P*_) is as follows. For *n* = 10, *n*_*P*_ in {2, 4, 8}, for *n* = 25, *n*_*P*_ in {2, 4, 8, 16} and for *n* = {50, 100, 250, 500, 1000, 2500, 5000, 10000}, *n*_*P*_ in {2, 4, 8, 16, 32}.

The metrics used to measure the performance of the developed heuristics are described in Table 2.

**Figure 5:**
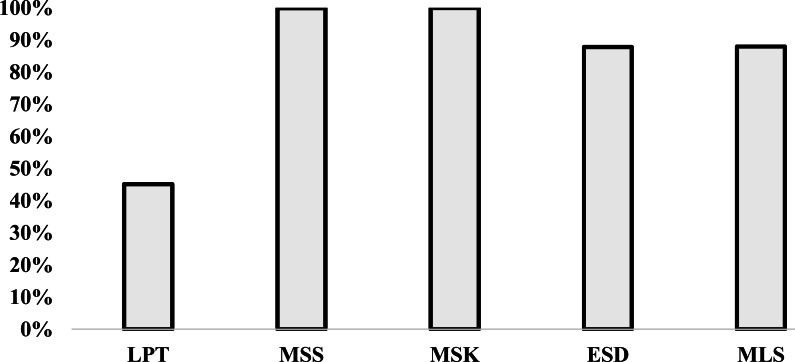
Variation of the percentage *Min* for all heuristics.

The variation of the percentage *Perc* is illustrated in Fig. 5. This figure is based on the results given in Table 3 in line *Perc* and each column *Min* for each heuristic. Figure 5, shows that the best heuristic from the literature is *MSK* and the best-proposed heuristic is *MLS*. The percentage that we have the minimum value compared with the best heuristics value for *MLS* is 87.9%. However, the percentage for the *MSK* is 100%. The difference between *MLS* and *MSK* is only 12.1%. This means that the results given by *MLS* are close to results obtained by *MSK*.

**Table 2:** Notation and metrics description.

Notation	Description
*Min*	The number of instances that the studied heuristic equals the minimum value given by comparing all heuristics.
*U*	The studied heuristic
$\hat {L}$	The maximum value of all lower bounds given in the literature review.
$MG= \frac{U-\hat {L}}{\hat {L}} \times 100$	gap between lower bound and the studied heuristic
*MGP*	The average of *MG*
*Opt*	The number of instances that the studied heuristic is equal to $\hat {L}$. This means that the number of instances in that we have the optimal solution is just when we calculate the studied heuristic.
*Time*	The spent time to execute the heuristic in corresponding instances. This time will be in seconds and we denote by “-” if the time is less than 0.001 s.
*A*.*Time*	The average of *Time* for a given set of instances
*M*.*Time*	The maximum of *Time* for a given set of instances
*Perc*	The percentage

**Table 3:** Heuristics comparison for the overall instances and classes.

	*LPT*		*MSS*		*MSK*		*ESD*		*MLS*	
	*Min*	*Opt*	*Min*	*Opt*	*Min*	*Opt*	*Min*	*Opt*	*Min*	*Opt*
*Total*	1061	1053	2346	2284	2349	2287	2063	2052	2066	2055
*Perc*%	45.1	44.8	99.8	97.2	100.0	97.3	87.8	87.3	87.9	87.4
*A*.*Time*	–		0.941		1.307		**0.002**		**0.002**	
*M*.*Time*	0.003		6.572		8.372		**0.007**		**0.006**	

**Notes.**

Remarkable values are in bold.

Table 3 presents the comparison between 2,350 instances for all heuristics. We denoted by *Total* and *Perc* the number of instances and the percentage, respectively, among the 2,350 instances of corresponding *Min* and *Opt*.

On the other hand, it is interesting to see that the *MSK* heuristic is more time-consuming as the average time is 1.307 s and the maximum time is 8.372 s. For the proposed heuristic *MLS* the average time is 0.005 s and the maximum time is 0.006 s. For the proposed heuristic *ESD* the average time is 0.002 s and the maximum time is 0.007 s.

Therefore, from Table 3 we can deduce that by adopting the difference of only 12.1%, we can choose the *MLS* heuristic instead of the *MSK* to have an acceptable approximate solution with minimum time. Indeed, the execution of the overall instances (2,350) costs 3,051.442 s for *MSK*. However, the same instances costs only 4.068 s, so we win 3,047.374 s.

From Table 3, we know here after the choice of the *MLS* impact only 12.1% of performance instances compared with *MSK*. This latter table shows that the proposed heuristics *ESD* and *MLS* reach the optimal solution in 87.3% and 87.4% cases, respectively.

For more details, Table 4 preset the percentage among the 470 instances of corresponding *Min* and *Opt* for each class.

Let us now give a time study to compare the literature heuristics and the proposed ones. Table 5 demonstrates that the maximum time of 8.372 s is obtained for the heuristic *MSK* when *n* =10,000. However, when *n* =10,000, the maximum time for *MLS* is 0.005 s which is less than the minimum value of *M*.*Time* for *MSK* (0.772 s).

Table 6 shows that for *MLS* the time is almost between 0.001 s and 0.006 s. However, for *MSK* the minimum value of *M*.*Time* is 6.490 s when *n*_*P*_ = 8 and the maximum value is 8.372 s when *n*_*P*_ = 32.

**Table 4:** Heuristics comparison total instances (*n*, *n*_*P*_) by classes.

	*LPT*		*MSS*		*MSK*		*ESD*		*MLS*	
	*Min*	*Opt*	*Min*	*Opt*	*Min*	*Opt*	*Min*	*Opt*	*Min*	*Opt*
Class 1	71.9	71.5	100.0	98.1	100.0	98.1	**90.2**	**89.8**	**90.6**	**90.2**
Class 2	41.7	40.6	99.8	96.2	100.0	96.4	87.2	86.6	87.2	86.6
Class 3	44.3	44.3	100.0	98.9	100.0	98.9	87.2	87.0	87.2	87.0
Class 4	54.9	54.9	99.6	94.7	100.0	95.1	88.3	87.4	88.5	87.7
Class 5	13.0	12.8	99.8	98.1	99.8	98.1	86.0	85.7	86.0	85.7

**Notes.**

Remarkable values are in bold.

**Table 5:** Behavior of the maximum time according to *n*.

*n*	*LPT*	*MSS*	*MSK*	*ESD*	*MLS*
10	–	0.005	0.082	**0.002**	**0.001**
25	–	0.017	0.252	**0.002**	**0.002**
50	–	0.038	0.587	**0.007**	**0.004**
100	–	0.075	1.184	**0.004**	**0.005**
250	–	0.097	0.772	**0.004**	**0.004**
500	–	0.231	0.893	**0.004**	**0.005**
1000	–	0.876	1.451	**0.004**	**0.005**
2500	0.001	2.357	1.960	**0.004**	**0.006**
5000	0.001	2.071	3.299	**0.004**	**0.006**
10000	0.003	6.572	8.372	**0.005**	**0.005**

**Notes.**

Remarkable values are in bold.

**Table 6:** Behavior of the maximum time according to *n*_*P*_.

*n*_*P*_	*LPT*	*MSS*	*MSK*	*ESD*	*MLS*
2	0.001	5.376	7.176	**0.002**	**0.001**
4	0.003	5.418	7.218	**0.002**	**0.001**
8	0.001	4.690	6.490	**0.002**	**0.002**
16	0.002	4.758	6.558	**0.004**	**0.004**
32	0.002	6.572	8.372	**0.007**	**0.006**

**Notes.**

Remarkable values are in bold.

In Table 7, we present the behavior of *M*.*Time* according to *Class*. Based on the results shown in this table, for *MSK* the maximum value of *M*.*Time* is 12.786 s for class 5. On the other hand, the maximum value for *MLS* is 0.006 s for all classes. It is clear that for *MSK* class 5 is harder than other classes. This is not the case applying the *MLS* heuristic.

**Table 7:** Behavior of the maximum time according to *Class*.

*Class*	*LPT*	*MSS*	*MSK*	*ESD*	*MLS*
1	0.003	4.448	6.248	**0.006**	**0.006**
2	0.003	6.756	8.556	**0.008**	**0.006**
3	0.005	7.122	8.922	**0.008**	**0.006**
4	0.002	5.933	7.733	**0.008**	**0.006**
5	0.003	10.986	12.786	**0.008**	**0.006**

**Notes.**

Remarkable values are in bold.

## Conclusion

This work developed an innovative procedure to introduce new heuristics for the identical parallel 2^*k*^ processors in different servers into the network. The procedure is articulated on the subdivision of the initial problem into multiple two sub-problems. Each problem is solved using a subset-sum problem. The generation of the full tree for each instance made the execution time less consuming compared with those in the literature. Almost 87.9% of the total sample is solved using the new heuristic. Its performance is almost the same as the best-known heuristics in literature. The performance of the proposed procedure is based on the running time. Indeed, we can solve big-scale instances in a remarkable running time. The experimental results show that we can gain 3,047.374 s when adopting the proposed procedure instead of those used in the literature. For future work, the used procedure can be utilized in an evolutionary meta-heuristic to enhance the results. In addition, the proposed procedure can be utilized to be applied to several scheduling problems. The proposed algorithms in this work could be utilized to cloud computing and developed for a load balancer using virtual machines in AWS or AZURE. A generalization of the proposed problem can be studied. This generalization is based on the consideration of the number of processors that are not in the power of 2.

##  Supplemental Information

10.7717/peerjcs.1408/supp-1 Supplemental Information 1 Code

10.7717/peerjcs.1408/supp-2 Supplemental Information 2 Code for ESD algorithm

10.7717/peerjcs.1408/supp-3 Supplemental Information 3 Instances of data used in the experimental results

10.7717/peerjcs.1408/supp-4 Supplemental Information 4 Code for MLS algorithm

## Additional Information and Declarations

### Data Availability

The following information was supplied regarding data availability: The codes are available in the Supplemental Files.
